# Views of healthcare providers on the provision of continuous care during labour by maternity care assistants in the Netherlands: a qualitative study

**DOI:** 10.1186/s12884-026-09630-z

**Published:** 2026-07-10

**Authors:** Amber J. C. Scheenen, Karina V. Chaibekava, Laurance Timmers, Hubertina C. J. Scheepers, Marianne J. Nieuwenhuijze

**Affiliations:** 1 Department of Obstetrics & Gynecology, Maastricht University Medical Centre (MUMC+), Maastricht University, Peter Debyelaan 25, Maastricht, 6229 HX The Netherlands; 2 School for Oncology and Reproduction (GROW), Maastricht University, Peter Debyelaan 25, Maastricht, 6229 HX The Netherlands; 3 Obstetrician, Department of Obstetrics & Gynecology, Maastricht University Medical Centre (MUMC+), Maastricht University, Peter Debyelaan 25, Maastricht, 6229 HX The Netherlands; 4 Research Centre for Midwifery Science, CAPHRI, Maastricht University, Universiteitssingel 60, Maastricht, 6229 ER The Netherlands

**Keywords:** Parturition, Childbirth, Obstetrics, Obstetric pain, Labour pain, Analgesia, Obstetrical, Continuity of care, Implementation science

## Abstract

**Background:**

Continuous care during labour has proven to be beneficial in reducing the need for medical intervention, such as epidural anaesthesia and caesarean sections, but is yet to be implemented on a large scale. The Continuous Care Trial looked at effectivity and costs of continuous care by maternity care assistants. Complementary to the Continuous Care Trial and in the interest of implementation, this study aims to explore the experiences of involved healthcare providers.

**Methods:**

This is a descriptive qualitative study. Data were collected through individual interviews and focus group discussions based on a semi-structured interview guide with 17 healthcare providers involved in the provision of continuous care. Manifest content analysis was conducted using the methodology of Graneheim and Lundman to extract categories from the data.

**Findings:**

Three main categories were identified: ‘experiences with continuous care by maternity care assistants’, ‘perceived value of continuous care’ and ‘suggestions for further implementation’. Participants were positive about the provision of continuous care by maternity care assistants. Reservations about the added value of continuous care depended on professional background and setting. Specific aspects for improvement were training and the collaboration within hospital setting.

**Discussion:**

The findings advocate for an expansion of preparatory and evaluative activities when implementing continuous care, as acceptance of the intervention by healthcare providers is a major determinant for the implementation, especially in hospitals. The findings of this study are of broader interest internationally, given the rising interest in feasible ways to implement continuous support during birth.

**Conclusion:**

This study highlights the necessity of improvements in education and collaboration in hospitals to enhance acceptance of implementation of continuous care during labour.

**Supplementary Information:**

The online version contains supplementary material available at 10.1186/s12884-026-09630-z.

## Background

With the increase in medical knowledge over the past decades, interventions such as anaesthesia and caesarean sections have become available to alleviate pain and improve maternal and neonatal outcomes when necessary [1]. However, these interventions are associated with complications and increased medical costs [2–5]. There are concerns that current increasing rates of non medically indicated interventions during childbirth are associated with increased rates of perinatal and maternal morbidity or mortality [6]. For these reasons, interest in exploration of alternative methods of labour support to improve both women’s experience of childbirth and maternal and neonatal outcomes has grown.

The Continuous Care Trial (CCT) is a recent multicentre randomized controlled trial on continuous care during labour by trained maternity care assistants (MCA) in the Netherlands, testing the effects and costliness [7]. MCAs complete a vocational training (three years training to become senior nursing assistant and one additional year practice-oriented training to become MCA) and commonly assist the midwife from the moment of active pushing in low-risk births, and provide postnatal care in the first week. In this study, provision of continuous support was defined as nonstop intrapartum presence and informational, practical, physical and mental support, by one MCA, during midwife-led and obstetrician-led births from the start of labour till the start of active pushing. Continuous care encompasses nonstop presence and many forms of support, such as massaging, applying pressure to the back, offering warm or cold cloths, active coaching or providing reassurance and offering additional explanation, and is tailored to the needs of the woman [7, 8]. Women were invited during pregnancy and informed at 34 weeks gestation, during a standard home visit by the maternity care agency, about the definition and thus type of care they could request from the MCA. Involved MCA’s were additionally trained on provision of support, recognition of the changes in labour and need for further medical assistance and thereby the boundaries of their care [7]. The results of this study suggest that providing continuous care by trained MCAs improves vaginal birth rates and reduces the need for epidural or other analgesia and caesarean sections, without compromising patient safety or increasing peripartum costs [8].

Despite reducing the need for interventions during labour, improving maternal and neonatal outcomes [2, 9], and recommendations by the World Health Organization (WHO), continuous care is not common practice in maternity care in high-income countries [10, 11]. Implementation rates in the Netherlands are still low, approximately 37% in hospital in 2016 [12]. Midwives are unable to provide continuous care due to high workload in hospital and high case load in community care and due to difficulty of combining this care with medical responsibilities [13, 14]. The results of the CCT and other trials give reason to further explore implementation strategies for provision of continuous care by trained MCAs.

The implementation of continuous care can be conceptually guided by the five-stage implementation model by Grol and Wensing for translating healthcare innovations into routine practice [15]. Within this model, the first stage concerns the development of the proposed change, in this case the introduction of continuous support during labour provided by MCAs. The second stage focuses on identifying barriers and facilitators to implementation through a multilevel perspective encompassing six domains: the innovation, the professional, the patient, the social context, the organizational context, and the wider economic and political context [16]. Findings from this analysis subsequently inform the third and fourth stages, which involve the development and execution of tailored implementation strategies to address the identified barriers. The fifth stage consists of evaluation, follow-up, and refinement to support sustainable integration into routine care [15] (see Additional file 1).

This study is situated within the second stage and aims to generate a comprehensive understanding of the contextual determinants influencing implementation. To better understand barriers within the professional and social domains, a more in-depth exploration of healthcare providers’ experiences with continuous care, as well as perceived hindering and facilitating factors, is warranted [17]. Particularly on the perspective of healthcare professionals little has been reported [17].

The primary objective of this study is to explore the experiences of healthcare providers with continuous care during labour, with specific focus on the experience of MCA’s that delivered continuous care. The insights gained in this study may support further efforts in implementation.

This study is guided by the following research question: How was continuous care during labour by maternity care assistants experienced by healthcare providers?

## Methods

### Aim and design

This is a qualitative study, with manifest content analysis, using interviews to describe the experiences of healthcare providers with the provision of continuous care by MCAs, and subsequently explore facilitators and, in greater depth, barriers, which is useful in formulation of steps towards implementation [18, 19].

The medical ethical committee approved the CCT. For this qualitative study, an amendment was approved on 23-02-2022. All participants signed an informed consent.

Results are reported according to the Standards for Reporting Qualitative Research (SRQR) (see Additional file 2).

### Participants and setting

The healthcare providers included in the interviews were either MCAs who provided continuous care after training, and labour ward nurses and hospital- and community-based midwives who were present at one or more of the 208 births where continuous care by an MCA was provided within the CCT in the period 2018 until 2020.

Within the Dutch maternity care system, labour ward nurses are routinely present in hospital and assist the midwife or obstetrician during labour. Due to high workload and medical responsibilities in hospital, they do not routinely offer 1:1 support. Hospital-based midwives are involved in hospital births in obstetrician-led care. Indications to give birth in hospital with a hospital-based midwife include a request for epidural analgesia. Obstetricians are only involved in hospital births if further intervention is required, for example with a vacuum extraction or caesarean section. Community-based midwives work outside of the hospital and are present during home births, or may use hospital facilities (labour rooms) for women who prefer to give birth in a hospital building if there is no medical indication to give birth in obstetrician-led care. The maternity care assistant routinely assists the community-based midwife in the final stage of labour and provides care postnatally (See additional file 3).

Two maternity care agencies with whom the CCT cooperated, 20 midwifery practices in the south-eastern region of the Netherlands, and coordinators of hospital staff of the 3 hospitals that participated in the CCT were contacted via a recruitment letter or phone and asked to propose candidates for an interview. All proposed candidates were then approached by the researcher via phone or email. If interested in participating, they were informed about this study, consent was signed, and an appointment was made for the interview.

To gain a deep understanding of the perspective of MCAs who delivered continuous care, and a general understanding of the perspective of nurses and midwives, we strived to include 8 MCAs and 8 other professionals being community-based midwives, hospital-based midwives and nurses. Participant recruitment was continued till no new insights came up.

### Sampling strategy

To ensure diversity in interviewed MCAs, participant inclusion was guided by purposive sampling. The inclusion strategy intended to collect diverse and contrasting experiences, thereby enriching the data. Therefore the invitation outlined the following criteria for proposing candidates: had a positive/ negative experiences with continuous care; delivered continuous care during an exceedingly long/ short labour; accompanied the woman in labour from home to hospital whilst proceeding to deliver continuous care; prematurely ended the provision of continuous care on request; has little/much experience as an MCA.

For diversity among the nurses, midwives and hospital-based midwives, the invitation stated the following criteria: had a positive/ negative experience with continuous care; was involved in a labour with continuous care that proceeded slowly/ rapidly; has little/ much experience in maternity care.

### Data collection

The interviews were done either on site or online via Microsoft Teams, depending on the preference of the participant. Participants were asked to share their opinion on the provision of continuous care in an interview lasting 30–60 min. Questions in the semi-structured interview guide were based on the order of events in a birth with continuous care and consisted of open-ended questions (See Additional file 4). Data was collected from March till May 2022.

Additionally, due to high workload on the ward in all participating hospitals, nurses were approached on the ward of one participating hospital and asked to participate directly in a short interview, if they were not occupied with other activities. This approach resulted in two group interviews with respectively two and three nurses at the ward of one participating hospital.

### Data analysis

The interviews were audio-recorded, transcribed verbatim and analysed using qualitative date analysis software Dedoose version 9.0.46.

Data analysis was done continuously throughout the study while data collection was ongoing, with the aim of determining if no new information emerged or invitation of additional participants was needed.

To extract categories from qualitative data, content analysis was conducted using the methodology of Graneheim and Lundman [19, 20]. Transcripts were read to gain understanding of the overall content. Semantic units were highlighted in the text, abstracted and coded.

The codes were classified in sub-categories and subsequently combined to determine categories [19].

Quotes are used to illustrate the findings.

### Trustworthiness

To ensure rigor of this study, the Lincoln and Guba (1985) criteria were applied [21].

Credibility of this study was ensured in several ways. The research team consisted of professionals with varying perspectives and expertise, reducing risk of bias and improving internal validity. They were not involved in the care of the women. To establish prolonged engagement, one researcher conducted all interviews. Transcribed interviews were shared with the participants for a member-check. Furthermore, the participants in this study were from various professions and had experienced continuous care from various perspectives. Moreover, the recruitment letter stated criteria, to assure participants with varying experiences.

To facilitate transferability, an extensive description of the participant recruitment and characteristics was provided. The consideration of various perspectives from the involved professionals within the research team improve transferability of results to different contexts.

To limit dependability of the research, investigator triangulation was used. A medical student researcher and the first author independently coded the interviews. The chosen codes were discussed among the team to gain deeper understanding of the main categories.

Critical reflexivity was guaranteed by supplementation of reflexive notes in the data.

The interviews were conducted by the primary researcher. Due to limited experience and in order to assure good quality of the conducted interviews, the primary researcher was initially intensively supervised by the last author, who is experienced in qualitative research.

### Findings

Eight MCAs, five nurses, three community-based midwives and one hospital-based midwife were interviewed in this study.

Characteristics of the participants are presented in Table 1.

**Table 1:** Characteristics of 17 healthcare providers who participated in an interview

Candidate	Profession	Organization	Setting of the interview
MCA1	MCA	Maternity care agency	Individual
MCA2	MCA	Maternity care agency	Individual
MCA3	MCA	Maternity care agency	Individual
MCA4	MCA	Maternity care agency	Individual
MCA5	MCA	Maternity care agency	Individual
MCA6	MCA	Maternity care agency	Individual
MCA7	MCA	Maternity care agency	Individual
MCA8	MCA	Maternity care agency	Individual
CM9	Community-based midwife	Community midwifery practice	Individual
CM10	Community-based midwife	Community midwifery practice	Individual
CM11	Community-based midwife	Community midwifery practice	Individual
N12	Nurse	Hospital	Group
N13	Nurse	Hospital	Group
N14	Nurse	Hospital	Group
N15	Nurse	Hospital	Group
N16	Nurse	Hospital	Group
HM17	Hospital-based midwife	Hospital	Individual

*MCA* maternity care assistant, *N* nurse, *CM* community-based midwife, *HM* hospital-based midwife

### Categories

In the interest of implementation of continuous care, the experiences of healthcare providers with continuous care have been described in three categories (Table 2). These categories summarize the experiences with the provision of *continuous care by MCAs* within the context of the CCT, describe *the perceived value of continuous care* and define *suggestions for further implementation of continuous care.*

**Table 2:** Main categories and sub-categories

Categories	Sub-categories
Experiences with continuous care by MCAs	Training of the MCA
	Collaboration and task division with maternity care providers
	MCA as provider of continuous care
Perceived value of continuous care	The effect of continuous care
	Preference for elements of continuous care
	Added value of continuous care in various settings
Suggestions for further implementation	Practical challenges
	Points of improvement

*Abbreviations*: *MCA* Maternity care assistant

### Experiences with continuous care by MCAs

Healthcare providers reflected on their experiences with continuous care during labour in the context of the CCT.

#### Training of the MCA

Regarding the preparation of the MCAs for the provision of continuous care, many of the MCAs and a community-based midwife agreed that the training prior to the trial was sufficient and should be incorporated in further implementation of continuous care as it was of added value. However, feedback from the nurses and hospital-based midwife suggested preparation of the MCAs could be improved, and some MCAs mentioned that the training could be more detailed. Suggestions for improvement in the training included items such as, knowledge on when to contact other staff, about the roles and responsibilities of other healthcare providers, and on the recognition of stages of labour. Additionally, it should include skills on how to be more assertive, take initiative and act in advance rather than to respond to situations as they occur and to offer suggestions rather than to expect women to ask for help and make a strong first impression. Some MCAs mentioned that they deepened their knowledge during the trial period by exploring cases from colleagues and reading about experiences of others on the internet.

Prior experience with childbirth played a key role in the preparation and confidence of the MCA. A suggested addition to the training of the MCAs included learning from a more experienced MCA or other maternity care provider. Experienced MCAs could accompany and supervise unexperienced MCAs when providing continuous care for the first time.

#### Collaboration and task division with maternity care providers

Continuous care means a new division of tasks among healthcare providers, therefore collaboration between various maternity healthcare providers still had to be established at the beginning of the trial. There was little experience with continuous care and lack of knowledge on the benefits of the intervention. *‘…it was not yet clear what our role and tasks were. I soon felt*,* …*,* they think I’m not of added value.’* (MCA1).

In community-based care, midwives and MCAs unanimously agreed there was effective communication, proper transfer of medical information and satisfying task division between midwives and MCAs through constant communication. Midwives acknowledged trust in the capabilities of MCAs grew over time. In hospital on the other hand, several MCAs and nurses mutually expressed their critique as there was poor integration of the MCAs with hospital staff, resulting in little collaboration, communication, or transfer of medical information. Issues arose due to lack of information, lack of faith in continuous care, lack of teambuilding and poor communication. Nurses said that they were poorly informed about the tasks of the MCA in their setting, an appreciated task was unwantedly taken from them, *‘I enjoy supporting women and that is what they are taking over. That is too bad.’* (N12) The hospital-based midwife said she could direct the MCA and her tips were appreciated, however nurses said they found it difficult to address the MCAs and communicate about task division as MCAs did not feel a part of the hospital team in their opinion. All healthcare providers agreed the MCAs did recognize the limitations of their responsibilities in hospital.

#### MCA as provider of continuous care

Healthcare providers agreed that the experience and care background of the MCA were of importance in the provision of continuous care. MCAs were able to recognize the necessity of medical staff or interventions, and knew when to take a step back, even if this meant discontinuing their care. The confidence of the MCA to answer initial questions of the parents, and the capability to handle emergency situations was acknowledged by nurses. However, the nurses mentioned that not all MCAs were suitable providers of continuous care. They emphasized that women often do not know what to expect during labour and therefore do not know what to ask for. Therefore, provision of continuous care required that MCA observe, assess and proactively anticipate to the needs of the woman and her partner by taking initiative and establishing effective communication. This requires being confident, creating a pleasant atmosphere, showing empathy, having a sense of responsibility, knowing when to ask for help and remaining respectful and calm in stressful situations. When asked about the potential provision of continuous care by medical or midwifery students, participants expressed concerns around lack of experience or confidence in students.

Nurses suggested continuous care to be more approachable if women were acquainted with the provider prior to labour. However, midwives and MCAs agreed that earlier involvement of the MCA for continuous care during labour already allowed for a better introduction of the MCA to the woman in labour compared to care-as-usual, in which the MCA is only introduced in the final stage of labour. Participants suggested exploration of this issue among women.

### Perceived value of continuous care

Considering further implementation, best practice of continuous care was described, based on the experienced effect, preference for elements of continuous care and the added value of continuous care in various settings.

#### The effect of continuous care

MCAs, the hospital-based midwife, and the community-based midwives acknowledged the appreciation of parents, as they experienced that women felt safe, calm, well prepared, able to make well-informed decisions and able to maintain focused, as a result of the continuous reassurance offered by the MCA. Especially when maternity healthcare providers were called away, when attending multiple births at once, *‘… left alone*,* in so much pain*,* [parents] doubted if what was happening is normal or not*.’ (MCA5).

As partners were actively involved in the birth by the MCA, healthcare providers noted that *‘[Partners appreciated] to make small talk*,* to not only be focused on the contractions. Because often there is tension in a labour room.*’ (MCA1) The assured state of the partner and pleasant atmosphere was also considered favourable for the woman in labour.

While MCAs explained it was their role to include the partner, nurses expressed concerns about the role of the partner being taking over by the MCA. Nurses also expressed concerns about the negative effects of continuous care, as the addition of an MCA was distracting for some women and sometimes the MCA created false hope by misinforming the woman about her stage of labour.

All MCAs unanimously described the improvement continuous care offered to their profession. It was noted that the positive outcomes of continuous care, the privilege of being present during labour and the joy MCAs had in offering continuous care outweighed the additional burden and hours of its provision. A midwife also noted the improved connection between midwife and MCA as a result of their earlier deployment.

#### Preference for elements of continuous care

Participants stressed that preference varied, but presence rather than the content of the continuous care provided by the MCA, seemed of significant importance to most women. The findings suggest the strength of the intervention lies within its apparent simplicity. ‘*Someone being present*,* what she does is not of much importance.’* (CM11) Additionally, the MCA had an essential role in the provision of information on what is happening and what is yet to come. Towards the end of the birth women seemed most helped by verbal coaching.

#### Added value of continuous care in various settings

Healthcare providers stressed the added value of the MCA for both woman and partner, but also nurses and midwives. The added value of the MCA in community-based care was greatest if the midwife was occupied with multiple labours.

As it is even more common for nurses and hospital-based midwives to be occupied with multiple labours, women were more often left alone in hospital. Additionally, the importance of continuous care was expected to be greater in hospital due to easy access to epidural or other analgesia. The hospital-based midwife explained, offering epidural anaesthesia was sometimes also considered beneficial to hospital staff when they were very occupied. *‘But in hospital care I think it is definitely of added value to have someone present continuously*,* to make sure other means [of analgesia] are not as easily considered*,* just because the nurse is busy and the supervisor*,* like midwife or resident*,* are busy.’ (HM17)*.

Nurses acknowledged the possible added value of continuous care in hospital but were sceptical about its current form within the CCT. From their perspective continuous care by the MCA was often superfluous and a more proactive attitude of the MCA would be of added value.

### Suggestions for further implementation

Participants offered suggestions for further implementation of continuous care.

#### Practical challenges

Healthcare providers expressed their worries about the planning of the extra hours required to provide continuous care and the logistics of continuous care. Midwives responded they sometimes found it challenging to decide when it was best to call the MCA.

Additionally, various healthcare providers expressed their reservations regarding the protocolled ending of MCAs’ continuous care. It felt unnatural to leave once the placenta was born after being involved in the birth for many hours. In practice, MCAs left one or two hours after the placenta, after further assisting hospital staff, taking care of the woman, and being able to say goodbye. Inconsistency in the ending of continuous care, led to misunderstandings between healthcare providers in hospital. If an epidural was placed, the MCA explained they preferred to stay till the woman was completely pain free or during the remainder of labour. However, midwives and nurses commented in their opinion it was ineffective if the MCA stayed after an epidural was placed, as there was little to do, possibly for many hours.

#### Points of improvement

Healthcare providers suggested improvements in provision of information on continuous care. Active promotion of continuous care is still needed in the hospital, including better education of hospital staff on the goals of continuous care and tasks of the MCAs. Furthermore, a midwife doubted if women understood that they could stop continuous care at any moment and stressed to emphasize the freedom of choice women have throughout labour when accepting continuous care.

Nurses and MCAs mutually agreed it would be of added value to the collaboration in hospital if they got more acquainted. Nurses did not feel acquainted with the MCA, MCAs felt their role in hospital was not well understood and the hospital-based midwife felt the introduction of the MCA towards the women and partners could be improved. It was suggested to enter the room together. The midwife or nurse would then introduce the MCA. Furthermore, nurses and midwives expressed their interest in a group meeting, when reintroducing continuous care.

*‘I think the collaboration between MCA and the maternity ward in hospital could be improved*,* in order to jump in more easily to meet each other’s needs. In order to enter the room as a team …’ (N14)*.

Regarding logistics, it was suggested to include the wish to receive continuous care in the electronic patient file, pregnancy folders and birth plan.

### Discussion

In this study, we explored experiences with the provision of continuous care during labour from the perspectives of MCAs delivering this care, as well as other healthcare professionals involved in labour where continuous care was provided. Guided by the second phase of the Grol and Wensing implementation model, we explored barriers and facilitators at the level of individual healthcare professionals and within their social and organisational work environment. This study is concerned with gaining insight into the factors that may influence successful implementation, thereby providing the foundation for the development of targeted implementation strategies [16].

As implementation of continuous care during labour by MCAs was first examined in the context of the CCT, feedback from participants of the trial is valuable for further implementation. Positive changes in the profession of MCA were noted. It was suggested training of the MCA could be deepened, and collaboration could be improved in hospital care. It seems that implementation of continuous care in community-based care is of most added value if the midwife is busy with multiple births. Continuous care always seemed of added value in the hospital, provided that collaboration between MCAs and hospital staff is improved in hospital care.

The findings on hindering factors and points of improvement offer steps towards implementation. Hindering factors in implementation are adequate planning of the extra hours of care by MCAs and logistics regarding registration of women for continuous care within their medical file. Furthermore, difficulty was noted in starting up and ending continuous care. Reconsideration of the conditions to start and end continuous care is indicated, especially if an epidural is placed. Other points of improvement include spread of information among both healthcare providers and women, and organizing a meeting among healthcare providers to improve acquaintance and collaboration.

This study described the challenges that are faced in the subsequent acceptance phase of the implementation model of Grol and Wensing [16]. In order for healthcare providers to integrate evidence-based practice in their care, proper information and motivation are required [16]. Often lack of agreement, motivation and little trust in the positive outcomes of an intervention result in tension among healthcare providers and poor integration of evidence-based care [16, 22]. Poor provision of information in hospital and misunderstanding on the role of the MCA led to resistance of hospital-based healthcare providers and limited collaboration between MCAs and nurses. Unwanted changes in task division and potential increase of workload for other hospital staff were attributed to a lack of training and knowledge of the MCAs [22].

The findings of this study advocate for the expansion of the preparatory and evaluative activities in the implementation of continuous care. As described by Brüggemann, Ebsen (2014) [22], training on continuous care should also include training on the integration of continuous care for all maternity care providers as their acceptance is a major determinant in the extend of implementation in the hospital. Such training includes providing information on the purpose and importance of continuous support and offering suggestions to facilitate effective collaboration. Regarding evaluation, these findings advocate for organisation of meetings to discuss collaboration and progress.

### Complementary findings

The aim of this study was to explore experiences and subsequently identify barriers in order to facilitate implementation. As barriers and areas for improvement were considered particularly valuable for informing the development of steps toward implementation, these aspects are examined in greater depth. Positive outcomes of continuous care were also identified; however, these were not explored extensively in the present paper, as complementary studies reporting the findings of the randomized controlled trial and participants’ experiences have been published separately and also address the added value of continuous care. Therefore, the primary contribution of the present study lies in its more detailed exploration of barriers relevant to implementation.

The inclusion of both MCAs as providers of continuous care and other maternity care professionals involved in labour where continuous support was provided was intentional. The study population comprised eight MCAs, the primary providers of continuous care, and nine healthcare professionals involved in its provision, thereby capturing both direct caregiving experiences and involvement in the care process.

This study therefore provides complementary findings on the *provision* of continuous care to women’s experiences of *receiving* continuous care during labour. Qualitative data on women’s experiences of the Continuous Care trial were collected in a separate study [23].

### Future perspectives

Relevant for future implementation are the finding that MCAs expect a rise in interest for the profession of MCA. This is favourable as Dutch maternity care agencies are facing shortages [24].

### Strengths and limitations of this study

As MCAs are employed in most regions of the Netherlands, the described experiences with continuous care and points of improvement are of interest for implementation in other Dutch regions.

Results of the CCT and points of improvement concerning continuous care are also of interest internationally, due to the rising interest in the use of MCA-type profession in hospital labour wards or doula care in other high-income countries, where similar issues can hinder implementation [25]. The findings of this study are applicable in part to maternity care outside of the Netherlands, due to the unique setup of maternity care in the Netherlands with MCAs. It is relevant to distinguish between doula care and care provided by MCAs, as well as to recognise the added value of the MCA within the clinical setting. Differences include accessibility, which is limited for doula care by high private costs, lack of insurance coverage, and limited workforce diversity, resulting in disproportionate use among primarily white, middle- and upper-class women [26]. Moreover, in the Netherlands MCA’s are an accepted and trained group within the obstetrical work field as they complete a vocational training of three years to become a senior nursing assistant and one year to qualify as an MCA. The quality of this training is guaranteed by government [25]. Nevertheless, the results can be translated to international contexts, as there is a broader global demand for continuous labour support and maternity care in many countries already incorporates various forms of labour assistance [27, 28].

Despite criteria in the invitation to assure diversity, as participation in this study was voluntary, it is likely those with a positive experience more willing to participate. Additionally, as included maternity care providers accompanied multiple births during the trial, they often meet more than one inclusion criteria. The combination of positive and critical remarks that have emerged in the interviews suggest that consideration of the inclusion criteria has led to a diverse study population. However, aside from diversity in years of experience and profession, the diversity among the other inclusion criteria is not exactly known. Despite the intention to include staff from all 3 participating hospitals, for this study only staff from one hospital has been included. Staff from the other hospitals were too busy. As a result, statements made by nurses and the hospital-based midwife represent the perspective from one hospital, still we have no reason the assume that the situation was different in the other hospitals.

Due to a wide range in years of experience within the profession, the effect of years of experience within maternity care on a participant’s point of view on continuous care is minimized.

## Conclusion

Feedback on the provision of continuous care during labour by MCAs in the context of the Continuous Care Trial is valuable for further implementation of continuous care. Based on the findings of this study it is expected that the profession of MCA will gain interest due to the addition of the highly appreciated task of providing continuous care during labour. The addition of continuous care by suitable MCAs, preferably with wider experience, is of greatest value in busy community-based practices and all hospitals. Education on beneficial effects of continuous care and agreement on task division and collaboration between healthcare providers within hospitals need to be improved to encourage acceptance and implementation.

## Supplementary Information

Supplementary Material 1.

Supplementary Material 2.

Supplementary Material 3.

Supplementary Material 4.

## Data Availability

The datasets used and/or analysed during the current study are available from the corresponding author on reasonable request.

## Abbreviations

CCT: Continuous Care Trial
MCA: Maternity Care Assistant
