# Supplementary material for: Views of healthcare providers on the provision of continuous care during labour by maternity care assistants in the Netherlands: a qualitative study
Source: BMC Pregnancy Childbirth. 2026 Jul 10;26:938. doi: 10.1186/s12884-026-09630-z (PMC13499301; doi:10.1186/s12884-026-09630-z)
Supplement: Supplementary file 1 [file 12884_2026_9630_MOESM1_ESM.docx]

**Additional file 1. Illustrative overview of the applied theoretical framework**

The figure does not present original research data, study findings, or outcomes. This figure was generated using artificial intelligence solely for illustrative purposes. It is intended exclusively to visually depict the application of the Grol and Wensing implementation model as the theoretical framework underpinning the quantitative and qualitative research conducted in relation to the Continuous Care Trial. The framework and its relevance to the study are described in detail in the Background section of the manuscript.
