# Supplementary material for: Views of healthcare providers on the provision of continuous care during labour by maternity care assistants in the Netherlands: a qualitative study
Source: BMC Pregnancy Childbirth. 2026 Jul 10;26:938. doi: 10.1186/s12884-026-09630-z (PMC13499301; doi:10.1186/s12884-026-09630-z)
Supplement: Supplementary file 3 [file 12884_2026_9630_MOESM3_ESM.docx]

**Additional file 3. Organisation of maternity care in the Netherlands**

The Dutch maternity care system is divided in primary, secondary and tertiary care. Primary care is midwifery-led and community-based and encompasses low-risk pregnancies. Giving birth within primary care is possible at home, in birth centres or in hospital led by the community-based midwife. (De Vries et al., 2013) (Amelink-Verburg & Buitendijk, 2010)

Maternity care assistants (MCAs) undergo vocational training and typically support community-based midwives during the active pushing stage of low-risk births, as well as provide care during the first week postpartum.

The employment of maternity care assistants and the organisation of maternity and postnatal care are coordinated through maternity care agencies.

Secondary and tertiary care are obstetric-led and hospital-based and encompass intermediate or high-risk pregnancies. Within hospital women are looked after by hospital-based midwives, obstetrical nurses and medical doctors. (De Vries et al., 2013) (Amelink-Verburg & Buitendijk, 2010)

Initial postpartum care is provided by obstetrical nurses and transferred to the maternity care assistant supervised by a community-based midwife after hospital discharge.

The existing funding system in the Netherlands stipulates that community midwives receive compensation for each care episode provided to a client, covering pregnancy, labour and birth, and postpartum care. In contrast, hospitals are reimbursed according to 'diagnosis-treatment combinations (DTCs),' which are determined by the specific services, treatments, and procedures performed. (Nederlandse Zorgautoriteit, 2023).

**References:**

Amelink, M. & Buitendijk, S. (2010). Pregnancy and Labour in the Dutch Maternity Care System: What Is Normal? The Role Division Between Midwives and Obstetricians. Journal of midwifery & women's health, 55, 216-25.

De Vries, R., Nieuwenhuijze, M. & Buitendijk, S.E. (2013). What does it take to have a strong and independent profession of midwifery? Lessons from the Netherlands. Midwifery, 29(10), 1122-8.

Nederlandse zorgautoriteit. (2023). Beleidsregel prestaties en tarieven medisch specialistische zorg. Geraadpleegd op 29 maart 2024, van: https://www.nza.nl/zorgsectoren/medisch-specialistische-zorg/registreren-en-declareren/welke-regels-gelden-voor-de-medisch-specialistische-zorg-in-2023.
