# Supplementary material for: Views of healthcare providers on the provision of continuous care during labour by maternity care assistants in the Netherlands: a qualitative study
Source: BMC Pregnancy Childbirth. 2026 Jul 10;26:938. doi: 10.1186/s12884-026-09630-z (PMC13499301; doi:10.1186/s12884-026-09630-z)
Supplement: Supplementary file 4 [file 12884_2026_9630_MOESM4_ESM.docx]

**Additional file 4. Interview Guide Interviews Continuous Care Trial**

| **Topic** | **Opening** | **Continuation** | **Focus** |
| --- | --- | --- | --- |
| **Welcome and introduction** |  |  | Welcome, introduction attendees,  express thanks for participation,  invite participant to introduce themselves |
| **Introduction and explanation procedure** |  |  | Elaborate on goal of the study, emphasize confidentiality and respect, there are no incorrect answers |
|  |  |  | Procedural steps: (if not yet done) sign consent, elaborate on role of attendees, permission for recording, permission data processing, complete demographic data |
| **Involvement in CCT** | How were you involved in the provision of continuous care within the CCT/ in a labor where continuous care was provided? | What was your role?  What were your motives to provide continuous care?  What were your expectations? | Attitude/ expectations |
| **Experience with (the provision of) continuous care** | How was your first experience with (the provision of) continuous care? | What was positive about this experience?  What was negative about this experience?  Was this experience in line with your expectations? |  |
| **Organization at labor** | How did you experience being on call for the provision of continuous care?  OR  How was the organization at the start of labor and when starting up continuous care? | Was it clear to you how and when to call for continuous care/ you would be called for continuous care? |  |
|  | How was the start of labor and continuous care in your opinion? | How did you experience the moment of calling for continuous care, should this have been earlier or later?  Was there sufficient information exchange at this moment?  Did you encounter any problems at the start of labor? | Assure sufficient depth |
| **The effect of continuous care** | What was the added value of continuous care for the woman, in your opinion? | What effect did you notice of the continuous care you provided?  What was the added value for the partner?  Which type of continuous care was most effective in your opinion? | Elaborate on both positive and negative aspects |
|  | Which effect did (the provision of) continuous care have on yourself? | Did you encounter any problems?  How burdensome was (the provision of) continuous care on you? What was burdensome and what was of added value? |  |
| **Preparation of MCA** | How did the training prepare you for practice?  OR  Were the MCAS sufficiently prepared for the provision of continuous care, in your opinion? | Did the training prepare you sufficiently for practice?  How can the training be improved? |  |
|  | Did you prepare yourself in another way, aside from the training?  OR  Would you have liked to be prepared for a labor with continuous care? | Was there something you wish you knew about (the provision of) continuous care; did you encounter something for which you were not prepared? |  |
| **Future of continuous care** | How do you envision the implementation of continuous care? | Would you like to continue the provision of continuous care within your profession? *(if applicable)* Why yes/ why not? |  |
| **Closure** | Is there anything you would still like to share? |  | Thank again for participation |
